# Supplementary material for: Sex-Specific ADHD-like Behaviour, Altered Metabolic Functions, and Altered EEG Activity in Sialyltransferase ST3GAL5-Deficient Mice
Source: Biomolecules. 2021 Nov 24;11(12):1759. doi: 10.3390/biom11121759 (PMC8698374; doi:10.3390/biom11121759)
Supplement: Supplementary file 1 [file biomolecules-11-01759-s001.zip › biomolecules-1415288-supplementary.pdf]

Supplementary File (SF)

Materials and Methods

Animals

Two-three months-old male and female *St3gal5*$^{-/-}$ mice or C57BL/6 mice were used, mutant mice were bred as described elsewhere (Dukhinova et al., 2019, 2018; Sotnikov et al., 2013). Male mice were maintained individually in standard plastic cages (27×22×15 cm), and female mice were housed in groups of five per cage. Mice were housed on reversed 12-h light/dark cycle, under controlled laboratory conditions (22±1°C, 55% humidity, room temperature 22ºC, lights were on at 19:00), and food and water were available ad libitum. All efforts were undertaken to minimize discomfort and experimental protocols conformed to 2010/63/EU and were compliant with the ARRIVE guidelines (http://www.nc3rs.org.uk/arrive-guidelines). The experiments were approved by Hong Kong Government under individual license to conduct experiments using live animals and Animal Ethics Committee of School of Biomedical Sciences, Faculty of Medicine, the Chinese University of Hong Kong (Hong Kong).

Study design

A cohort of mice was studied in the open field test, elevated plus maze, dark/light box (n=10 in wild type groups and a group of *St3gal5*$^{-/-}$ males, n=6 for a group of female mutants; Fig S1A). Another cohort of mice was investigated in the 3-chamber sociability test, new object recognition test and the tail suspension test (n=8 per group; Fig. S1B). In a separate cohort of animals, mice were first examined for body weight and then they were used in the glucose tolerance test (n=9 mice per group). Additional groups (n=8 in each group) were challenged with i.p. injection of sterile saline (0.01ml/g) and 6 h thereafter were examined in the glucose tolerance test. The mRNA expression of IRA and IRB isoforms were carried out in the spleen, the cortex of the brain, and liver in separate

cohorts (n=3-5). The electroencephalogram (EEG) recordings were performed in separate cohorts (n=5 per group; Fig S1C). For further details on all protocols used, *see below.*

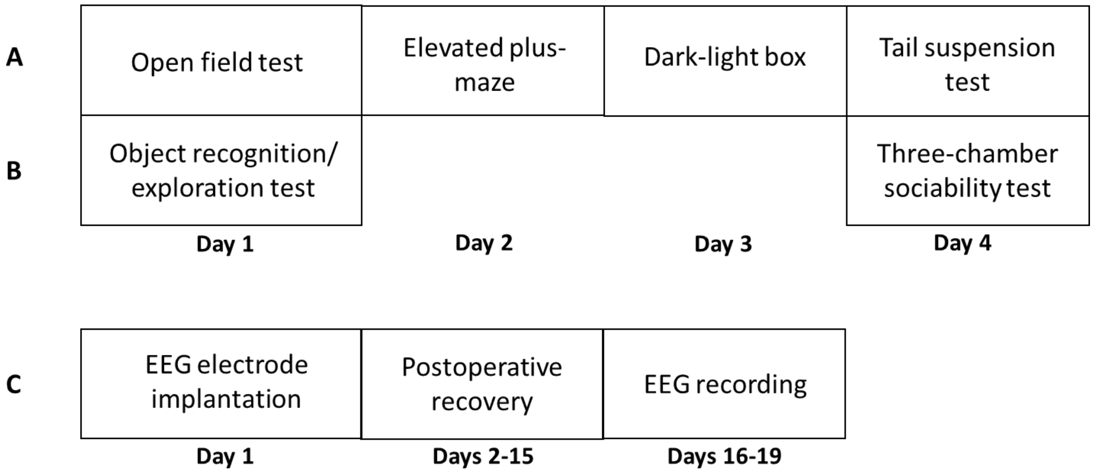

Figure S1. Experimental design of behavioral studies. **(A)** *St3gal5*$^{-/-}$ and wild-type mice of both sexes were tested in the open field test, elevated plus-maze, dark-light box and tail suspension test. **(B)** *St3gal5*$^{-/-}$ and wild-type mice were tested in object recognition/exploration test on day 1 and three-chamber sociability test on day 4. **(C)** In the separate cohorts of mice, electroencephalogram (EEG) recordings were performed after electrode implantation and postoperative recovery.

Behavioral tests

Behavioral tests were carried out during an active (dark) period of the animals' light cycle (07:00–19:00) and analyzed offline by the experimenter who was unaware of the genotype of each animal studied. One test was run per day. Mice of both genotypes were tested simultaneously. Behavioural equipment was thoroughly cleaned with water between each test.

Open field test

Mice were placed in the central arena of a customized square open field (40×40 cm), which was illuminated with a subtle light. Their behaviour was video recorded for 15 min as described elsewhere (Lim et al., 2016). Percent of time spent in the central zone (20×20 cm) and speed there

in, as well as the total distance travelled were analysed offline using Any-maze software (Anymaze, Dublin, Ireland).

Elevated plus maze test

The elevated plus maze consisted of four arms, 40 cm long and 5 cm wide, two of them were open, i.e., without walls and two arms were enclosed by 10 cm high walls. The maze was elevated 60 cm above the floor. Animals were placed on the central platform facing the open arm at the start of trial. Mean speed and time spent in open arms were scored using Any-maze software during 5 min under the illumination of 5 Lux, as described elsewhere (Lim et al., 2016).

Dark-light box test

The dark-light box test was adapted from a previously described method (Strekalova and Steinbusch, 2010; Veniaminova et al., 2020). The customized dark-light box consisted of two plexiglass compartments, a dark compartment, and a lit compartment of identical size (40×40×40 cm) that were connected by a tunnel. Mice were placed in the dark compartment and allowed to visit the lit box, which was illuminated by a 300 Lux light. The total time spent in the lit box were scored over 10 min.

Object recognition/exploration test

The apparatus consisted of a plastic cage (21×27×14 cm) with opaque walls and two identical objects: “brushes” or “flowers” (7×4×3 cm) placed symmetrically 2 cm away from the cage wall in the opposite corners of the cage, was lit with a light of illumination intensity 5 Lux (Strekalova et al., 2013; Veniaminova et al., 2017). Disposable objects “flowers” were made from paper and were changed to new once for each mouse; they were presented to mice on Day 1 during a 10-min test. Objects “brushes” were made of plastic and were washed with water and mild detergent; on Day 2

of the test, they were used to replace one of two “flower” objects. That way any contamination, such as the smell of a preceding mouse, was virtually excluded. As the duration of exploration behavior of *St3gal5*$^{-/-}$ mice was significantly decreased in comparison to controls, a ratio of new object recognition could not be assessed.

Three-chamber sociability test

The apparatus (60×40×22 cm) used for this test was divided by two removable partitions, which formed three chambers of the identical size (20×40×22 cm) (Ugo Basile S.R.L., Gemonio, Italy). Both partitions had a sliding door (4.0×3.5 cm), which could be lifted to form an entry between the chambers. Two metal cylinder-shaped cages were placed in the outer chambers. Mice were allowed to explore the central compartment for 5 min, the number of exploratory rearings was scored. As the doors to the tunnels were opened, mice were allowed to interact with another mouse, which was located in one of the cylinder-shaped cages or the other empty cylinder-shaped cages for 10 min. As general exploration and locomotor behaviour of *St3gal5*$^{-/-}$ mice was significantly impaired in comparison with controls, the assessment of sociability could not be performed.

Tail suspension test

Mice were subjected to a customized tail suspension system. Mice were suspended by their tail for 6 min under 25 Lux illumination as previously described (Malatynska et al., 2012; Veniaminova et al., 2020a). Immobility behaviour was defined as the absence of any movements of the animal’s head and body. The latency of the first episode of immobility and the duration of immobility were scored according to previously validated method of automated scoring.

Real-time reverse-transcriptase polymerase chain reaction (real-time RT-PCR)

The real-time qRT-PCR was performed using the SYBR Green master mix (Bio-Rad Laboratories, Philadelphia, PA, USA). qRT-PCR was performed in a 10μl reaction volume containing a SYBR

Green master mix (5 ul), RNase-free water (3 µl), specific forward and reverse primers used at the concentration 20 pmol/ul (1 µl) and cDNA (1 µl). The initial denaturation step for qRT-PCR was performed at 95°C for 5 min followed by 40 cycles of denaturation at 95°C for 30 seconds and annealing at 60°C for 30 seconds. The sequences of primers used are listed in *Table S1* (see below); The primers were purchased from Life Technologies (Carlsbad, CA, USA). All samples were run in triplicates.

Table S1. Primer sequence for mRNA expression analysis in mouse spleen, brain, and liver

Gene	Primer	Sequence
GAPDH	Forward	5'-ATGACCACAGTCCATGCCATC -3'
	Reverse	5'-GAGCTTCCCGTTCAGCTCTG -3'
IRA	Forward	5'- GGTTTTTGTCCCCAGGCCAT -3'
	Reverse	5'- GTGCTCCTCCTGACTTGTGG -3'
IRB	Forward	5'- CAATGGTGCCGAGGACAGTA -3'
	Reverse	5'- GTGCTCCTCCTGACTTGTGG -3'

Glucose tolerance test

Glucose tolerance test was performed as described elsewhere (Veniaminova et al., 2017, 2020). The test mice were fasted overnight for 18 h, beginning at 16:00. Next day, blood samples were obtained from the tail vein prior to glucose administration by oral gavage (2 g/kg, 1.8 g/l) at time point 0, and 5, 15, 30, 60, 120 minutes thereafter. The concentration of blood glucose was measured using the OneTouch UltraEasy glucometer and test strips (LifeScan OneTouch, Dubai, UAE) and the area under the curve (AUC) was calculated.

EEG recording and analysis

EEG recording was performed on freely moving male and female adult control and *St3gal5*$^{-/-}$ mice using a wireless transmitter and receiver system (BIOPAC Systems Inc, Goleta, CA, USA) as described previously (Kopeikina et al., 2020). To mount a wireless transmitter (size 10×12×8 mm) with two electrodes with the length of the wire 3 mm on a surface of the skull (EpochClass 2-channel EEG sensor, 2 mV, BIOPAC Systems Inc., Goleta, CA, USA, cat# EPTX-10128), the mice were deeply anesthetized with i.p. injection of ketamine/xylazine mix (ketamine dosage of 87.5 mg/kg and xylazine dosage of 12.5 mg/kg diluted in saline was administered at final volume of 0.1 ml per mouse) and placed on a stereotaxic holder, and an incision was made on their shaved scalp of the animal slightly behind the eyes along the midline of the length of 1 cm. Two holes (400 µm in diameter) were drilled at the position of 3 mm from bregma and lambda, above the hemispheres on the exposed scalp using a burr-type drill bit (Microtorque II, cat#10645, Ram Products Inc., NJ, USA), and the electrodes were inserted and attached to the scalp by cyanoacrylate glue (Evobond, Taiwan). The skin was sutured around the base of the transmitter, and the mice were placed in post-operation care cages for the observation. The mice were monitored for several hours postoperatively and then twice a day, and buprenorphine (0.05 mg/kg) was injected i.p. for pain management for 3 days postoperatively. The mice were allowed a 2 weeks recovery period prior EEG recording.

For the EEG recording, cages with individual mice were placed on a receiver platform connected with a computer. The 30 min EEG recording session was carried out daily from 8 pm to 9 pm during dark cycle of animals' cycle for 4 days. Recordings were analyzed using AcqKnowledge 4.4.2 acquisition software (http://www.biopac.com/product/acknowledge-software/, RRID: SCR_014279). The frequency of the high amplitude peaks, which were defined by the amplitude exciding the threshold of 10 µV, and the average amplitude (|Amax-Amin|) were calculated as described elsewhere (Dukhinova et al., 2018; Kopeikina et al., 2020).

Results

Supplementary tables with statistical results

Table S2. Two-way ANOVA results for statistical analysis of behavioral parameters[1]

Behavioral parameters						
Parameter	*Interaction, F*	*Interaction, p*	*Genotype, F*	*Genotype, p*	*Sex, F*	*Sex, p*
Total distance travelled in the open field	0.4426	0.5117	4.699	**0.0395**	0.04816	0.8280
Speed in the central zone of the open field	0.9741	0.3324	10.95	**0.0027**	0.4650	0.5011
Speed in open arms of the elevated-plus maze	2.334	0.1382	1.288	0.2664	0.05448	0.8172
Time spent in central zone of open field	0.003066	0.9563	7.857	**0.0093**	0.00011	0.9916
Time spent in open arms of elevated plus maze	2.852	0.1037	4.672	**0.0404**	1.065	0.3119
Time spent in lit box in the dark-light box	35.36	**<0.0001**	23.59	**<0.0001**	0.1620	0.6907
Duration of object exploration	9.814	**0.0052**	3.987	0.0596	3.569	0.0735
Number of exploratory rears in the three-chamber sociability test	0.09237	0.7643	48.33	**0.0001**	3.754	0.0669
Duration of immobility in the tail suspension test	1.542	0.2250	11.03	**0.0026**	1.095	0.3045

[1]F and p values are shown for interaction between genotype and sex, for genotype effect and for sex effect. Time spent in lit box of the dark-light box was decreased in *St3gal5*$^{-/-}$ females compared to wild-type females ($p<0.0001$, Tukey's test) and increased in wild type females compared to wild type males ($p=0.0003$, Tukey's test). Duration of object exploration was decreased in *St3gal5*$^{-/-}$ females compared to wild type females ($p=0.0084$, Tukey's test). Significant effects are in bold.

Table S3. Two-way ANOVA results for statistical analysis of expression of insulin receptor isoforms, metabolic parameters, and EEG-parameters[1]

Insulin receptor expression						
Parameter	*Interaction, F*	*Interaction, p*	*Genotype, F*	*Genotype, p*	*Sex, F*	*Sex, p*
IRA mRNA, spleen	22.55	**0.0005**	2.505	0.1395	19.33	**0.0009**
IRB mRNA, spleen	14.71	**0.0024**	6.109	**0.0294**	12.21	**0.0044**
IRA mRNA, brain cortex	0.1935	0.6678	1.742	0.2115	<0.0001	0.9999
IRB mRNA, brain cortex	0.7614	0.4000	1.492	0.2453	3.046	0.1065
IRA mRNA, liver	24.21	**0.0012**	0.1474	0.7111	3.774	0.088
IRB mRNA, liver	51.95	**<0.0001**	6.606	**0.0331**	0.07804	0.787
Metabolic parameters						
Body weight	5.377	**0.0232**	3.110	0.0820	28.39	**0.0001**
Basal glucose in naïve mice	0.5989	0.4444	0.1092	0.7431	1.157	0.2898
AUC for glucose tolerance test in naïve mice	0.03955	0.8435	0.7549	0.3910	21.36	**0.0001**
Basal glucose after i.p. injection challange	0.003750	0.9519	16.09	**0.0010**	0.00089	0.9765
AUC for glucose tolerance test i.p. injection challange	1.293	0.2722	8.849	0.0089	9.127	**0.0081**
EEG parameters						
Number of high-amplitude EEG-peaks	2.095	0.1671	74.56	**<0.0001**	0.7999	0.3844
Mean amplitude of the EEG-peak	3.236	0.0909	89.54	**<0.0001**	8.918	**0.0087**

[1]F and p values are shown for interaction between genotype and sex, for genotype effect and for sex effect. In comparison with wild-type males, IRA mRNA and IRB mRNA spleen expression were decreased in *St3gal5*$^{-/-}$ males and wild type females (IRA: p=0.0036, p=0.0002, IRB: p=0.0037, p=0.0011, respectively, Tukey's test).

IRA mRNA and IRB mRNA liver expressions were decreased in St3gal5$^{-/-}$ males and wild-type females when compared to wild-type males (IRA: p=0.023, p=0.006; IRB: p=0.047, p=0.005 respectively, Tukey's test). At the same time mRNA expression for IRB was significantly increased in St3gal5$^{-/-}$ females when compared to wild-type females and St3gal5$^{-/-}$ males (p=0.01, p=0.003, respectively, Tukey's test).

Body weight was higher in *St3gal5*$^{-/-}$ males than in wild type male and female groups (p=0.0328 and p<0.0001, respectively, Tukey's test). Significant effects are in bold.

References

Dukhinova, M., Kuznetsova, I., Kopeikina, E., Veniaminova, E., Yung, A.W.Y., Veremeyko, T., Levchuk, K., Barteneva, N.S., Wing-Ho, K.K., Yung, W.-H., Liu, J.Y.H., Rudd, J., Yau, S.S.Y., Anthony, D.C., Strekalova, T., Ponomarev, E.D., 2018. Platelets mediate protective neuroinflammation and promote neuronal plasticity at the site of neuronal injury. Brain. Behav. Immun. 74, 7–27. https://doi.org/10.1016/j.bbi.2018.09.009

Dukhinova, M., Veremeyko, T., Yung, A.W.Y., Kuznetsova, I.S., Lau, T.Y.B., Kopeikina, E., Chan, A.M.L., Ponomarev, E.D., 2019. Fresh evidence for major brain gangliosides as a target for the treatment of Alzheimer's disease. Neurobiol. Aging 77, 128–143. https://doi.org/10.1016/j.neurobiolaging.2019.01.020

Kopeikina, E., Dukhinova, M., Yung, A.W.Y., Veremeyko, T., Kuznetsova, I.S., Lau, T.Y.B., Levchuk, K., Ponomarev, E.D., 2020. Platelets promote epileptic seizures by modulating brain serotonin level, enhancing neuronal electric activity, and contributing to neuroinflammation and oxidative stress. Prog. Neurobiol. 188, 101783. https://doi.org/10.1016/j.pneurobio.2020.101783

Lim, L.W., Shrestha, S., Or, Y.Z., Tan, S.Z.K., Chung, H.H., Sun, Y., Lim, C.L., Khairuddin, S., Lufkin, T., Lin, V.C.L., 2016. Tetratricopeptide repeat domain 9A modulates anxiety-like behavior in female mice. Sci. Rep. 6, 37568. https://doi.org/10.1038/srep37568

Malatynska, E., Steinbusch, H.W.M., Redkozubova, O., Bolkunov, A., Kubatiev, A., Yeritsyan, N.B., Vignisse, J., Bachurin, S., Strekalova, T., 2012. Anhedonic-like traits and lack of affective deficits in 18-month-old C57BL/6 mice: Implications for modeling elderly depression. Exp. Gerontol. 47, 552–564. https://doi.org/10.1016/j.exger.2012.04.010

Sotnikov, I., Veremeyko, T., Starossom, S.C., Barteneva, N., Weiner, H.L., Ponomarev, E.D., 2013. Platelets Recognize Brain-Specific Glycolipid Structures, Respond to Neurovascular Damage and Promote Neuroinflammation. PLoS One 8. https://doi.org/10.1371/journal.pone.0058979

Strekalova, T., Anthony, D.C., Dolgov, O., Anokhin, K., Kubatiev, A., Steinbusch, H.M.W., Schroeter, C., 2013. The differential effects of chronic imipramine or citalopram administration on physiological and behavioral outcomes in naïve mice. Behav. Brain Res. 245, 101–106. https://doi.org/10.1016/j.bbr.2013.02.015

Strekalova, T., Steinbusch, H.W.M., 2010. Measuring behavior in mice with chronic stress depression paradigm. Prog. Neuropsychopharmacol. Biol. Psychiatry 34, 348–61. https://doi.org/10.1016/j.pnpbp.2009.12.014

Veniaminova, E., Cespuglio, R., Chernukha, I., Schmitt-Boehrer, A.G., Morozov, S., Kalueff, A. V., Kuznetsova, O., Anthony, D.C., Lesch, K.-P., Strekalova, T., 2020a. Metabolic, Molecular, and Behavioral Effects of Western Diet in Serotonin Transporter-Deficient Mice: Rescue by Heterozygosity? Front. Neurosci. 14. https://doi.org/10.3389/fnins.2020.00024

Veniaminova, E., Cespuglio, R., Cheung, C.W., Umriukhin, A., Markova, N., Shevtsova, E., Lesch, K.-P., Anthony, D.C., Strekalova, T., 2017. Autism-Like Behaviours and Memory Deficits Result from a Western Diet in Mice. Neural Plast. 2017, 1–14. https://doi.org/10.1155/2017/9498247

Veniaminova, E., Oplatchikova, M., Bettendorff, L., Kotenkova, E., Lysko, A., Vasilevskaya, E., Kalueff, A. V., Fedulova, L., Umriukhin, A., Lesch, K.-P., Anthony, D.C., Strekalova, T., 2020b. Prefrontal cortex inflammation and liver pathologies accompany cognitive and motor deficits following Western diet consumption in non-obese female mice. Life Sci. 241, 117163. https://doi.org/10.1016/j.lfs.2019.117163.
